#  Autogenous teeth used for bone grafting: A systematic review

**DOI:** 10.4317/medoral.22197

**Published:** 2017-12-24

**Authors:** Patricia Gual-Vaqués, Carlos Polis-Yanes, Albert Estrugo-Devesa, Raúl Ayuso-Montero, Antoni Marí-Roig, José López-López

**Affiliations:** 1 DDS, Master’s degree, School of Dentistry, University of Barcelona. University Campus of Bellvitge, Barcelona, Spain; 2 DDS, Professor of Master’s degree, School of Dentistry, University of Barcelona. University Campus of Bellvitge, Barcelona, Spain; 3 DDS, MD, PhD, Department of Odontoestomatology. School of Dentistry, University of Barcelona. University Campus of Bellvitge, Barcelona, Spain. / Dental Hospital University of Barcelona, (Barcelona University) / Oral Health and Masticatory System Group (Bellvitge Biomedical Research Institute) IDIBELL, Barcelona, Spain; 4 DDS, MD, PhD. Specialist in Maxillofacial Surgery, Head of Department of Maxillofacial Surgery, University Hospital of Bellvitge. Catalonia, Spain. / Oral Health and Masticatory System Group (Bellvitge Biomedical Research Institute) IDIBELL, Barcelona, Spain

## Abstract

**Background:**

Recently, bone graft materials using permanent teeth have come to light, and clinical and histological outcomes of this material have been confirmed by some studies. The aim of this systematic review was to evaluate the reliability of the autogenous tooth bone graft material applied to alveolar ridge augmentation procedures.

**Material and Methods:**

A systematic review of literature was conducted analyzing articles published between 2007 and 2017. The following four outcome variables were defined: a) implant stability b) post-operative complication c) evaluation of implant survival and failure rates, and d) histological analysis. A total of 108 articles were identified; 6 were selected for review. Based on the PICO (problem, intervention, comparison, outcome) model, the chief question of this study was: Can patients with alveolar ridge deficiency be successfully treated with the autogenous teeth used as bone graft?

**Results:**

The mean primary stability of the placed implants was 67.3 ISQ and the mean secondary stability was 75.5 ISQ. The dehiscence of the wound was the most frequent complication with a rate of 29.1%. Of the 182 analyzed implants, the survival rate was 97.7% and the failure rate was 2.3%. In the histological analysis, most of studies reported bone formation.

**Conclusions:**

There is insufficient evidence regarding the effects of autogenous teeth used for bone grafting to support any definitive conclusions, although it has been shown clinically safe and good bone forming capacity, and good results are shown about implant stability.

** Key words:**Bone regeneration, bone graft material, autogenous teeth graft, tooth.

## Introduction

Several techniques have been suggested for the regeneration of a deficient alveolar ridge segment in preparation for implant placement (1-6). The choice of the best graft material for each patient depends on many factors such as the anatomy, the morphology of the bone defect, type of prosthesis planned, and clinician and patient preferences (5).

Three properties are required for an ideal bone graft material: i) osteoconduction, which provides scaffolds for bone regeneration; ii) osteoinduction, which promotes the recruitment of bone-forming cells, such as undifferentiated cells and preosteoblasts, and formation of bone from these cells; and iii) osteogenesis, the induction of cells contained in the graft material to promote bone regeneration (7). Several bone graft materials have been used over time (8). Among them, autogenous bone is still considered the gold standard for bone augmentation because it exhibits all three properties (7-9). Nevertheless, it has some disadvantages including donor site morbidity and limited source (7-10), and also high resorption rates up to 50% (11). Other bone graft materials such as allografts, xenografts, and alloplastic bone grafts have been using over time, but they have some disadvantages (7-9). Allografts lack osteoproliferation and carry the risk of disease transmission, and xenografts and alloplasts only show osteoconduction (7). Therefore, development of an alternative graft material that surpasses all these limitations is expected.

Recently, bone graft materials using permanent teeth have come to light, and clinical and histological outcomes of this material have been confirmed by some studies (7-9,12). Tooth components are very similar to alveolar bone components. This leads to think about bone graft materials using the organic and inorganic components of extracted teeth (13,14). The total inorganic content of the enamel is 95%, while the organic content is around 0.6%, and water is 4%. In the dentin, however, the inorganic content is 70% to 75%, the organic content is 20% and, finally, water is 10%. When comparing these contents to alveolar bone, the organic, inorganic and water contents are 25%, 65% and 10%, respectively.

The inorganic material of teeth contains 4 types of calcium phosphate (hidroxyapatite, tricalcium phosphate, amorphous calcium phosphate, and octacalcium phosphate) (15). This inorganic content is known to have an osteoconductive property which makes it a biocompatible bone graft material. The organic matrix of dentine is predominated by a fibrous network of type I collagen that constitutes 90% of this content. The rest 10% of the dentin matrix is formed by non-collagenous proteins (osteocalcin, osteonectin, sialoprotein and phosphoprotein) which are involved in bone calcification, and growth factors, including bone morphogenetic proteins (BMP), LIM mineralization protein 1 and insulin-like growth factors. This gives teeth an osteoinductive property (8-10,16,17).

Autogenous tooth bone graft material (AutoBT) was first developed in 2008 and has been used mainly for guided bone regeneration to supplement dental implants (18). It is a bone graft material that is obtained using extracted teeth. The amount of bone graft obtained depends on the condition of discarded teeth and its histological outcomes are similar to autogenous bone grafts (19).

Dentin tooth can be classified into three groups according to the degree of demineralization; undemineralized dentin (UDD), partially demineralized dentin matrix (PDDM) (70% decalcified) and demineralized dentin matrix (DDM). Some papers have shown that UDD is less effective in bone formation whereas other studies have shown that DDM is biocompatible and also osteoinductive, similar to demineralized bone matrix (9). Koga *et al.* (9) concluded in their in vitro study that PDDM with large particle (1000µm) has much more bone regenerative activity in comparison to UDD. This could be explained because demineralization enhances the osteoinduction capacity of tooth material by exposing organic substances within the teeth to the surface, increasing porosity and surface area, and decreasing crystallinity (8). Nevertheless, some authors have reported successful bone regeneration applying UDD (20).

UDD can be easily obtained from a dentin grinder, after disinfection and cleaning process. PDDM can be only obtained from the Korea tooth bank after a partial demineralization process of the dentin (14). In any case, teeth must be free of restorations and caries, and endodontic teeth must be excluded.

The purpose of this systematic review was to assess the reliability of the autogenous tooth bone graft material applied to alveolar ridge augmentation procedures, in preparation for implant placement.

## Material and Methods

-Protocol

This review employs the PRISMA (Preferred Reporting Items for Systematic Review and Meta-Analyses) statement (21). A detailed protocol following the PICO system was designed to answer the following question: Can patients with alveolar ridge deficiency be successfully treated with the autogenous teeth used as bone graft? 

(P) Patient/problem: alveolar ridge deficiency 

(I) Intervention: autogenous teeth used as bone graft 

(C) Control: autogenous bone graft, allograft or xenograft

(O) Outcome: bone forming capacity/no bone forming capacity

-Selection criteria

An electronic search of English literature was carried out in January 2017 in Medline/PubMed, Cochrane and Scielo databases. Publications between 2007 and 2017 were included.

-Search methods

The combination of these keywords was used in the search: (extracted teeth AND autogenous graft) OR (autogenous tooth bone graft) OR (human dentin AND bone regeneration) OR (demineralized dentin AND bone regeneration) OR (tooth AND bone graft) OR (autogenous tooth bone) OR (autogenous teeth). As a result, 108 articles from Medline/PubMed were analyzed.

-Inclusion and exclusion criteria

The literature search was limited to dental journals published in English language. The inclusion criteria were in vivo studies, including at least 5 patients per study, wherein implants were reviewed from at least 6 months after being placed. Randomized and non-randomized clinical studies, cohort studies, case-control studies and case series have been considered, while case-reports have been dropped out. Studies involving patients affected by congenital malformations or tumors have been also excluded.

-Outcome variables 

The following four outcome variables were defined: a) implant stability [Ostell Mentor (Integration Diagnostics, Goteborg, Sweden)], b) post-operative complication c) evaluation of implant survival and failure rates, and d) histological and histomorphometric analysis.

-Data extraction

All headlines were screened in order to drop out irrelevant studies or animal and in vitro manuscripts. After that, abstracts were screened due to analyze the number of patients and basic characteristics of the study. The publications that remained after the abstract screening were analyzed according to inclusion/exclusion criteria. Finally, 6 articles were included in the present review.

A meta-analysis of the data reported in this systematic review could not be performed, due to the heterogeneity of the data of the manuscripts included.

-Quality of evidence

The table for the Oxford Centre for Evidence-Based Medicine (OCEBM) levels of evidence (Howick, Chalmers, Glasziou, Greenhalgh, Heneghan, Liberati, Moschetti, Phillips, Thornton, *et al.*) was used, and a level of evidence was assigned as established in the OCEBM introductory document (22). The 2011 OCEBM levels of evidence is a hierarchy of the likely best evidence, it consists of 7 questions to appraise evidence for prevalence, accuracy of diagnostic tests, prognosis, therapeutic effects, rare harms, common harms, and usefulness of (early) screening and 5 levels that rank articles according to the study design.

## Results

**Figure 1:**
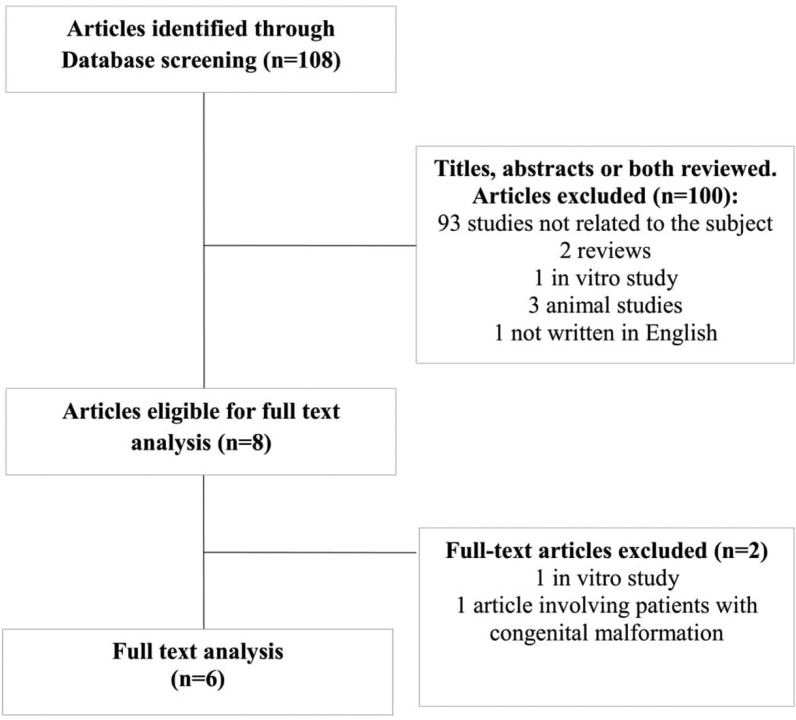
Flow of articles through the systematic review.

108 titles were obtained from the electronic search, ranging from 2007 up to 2017. The first screening of headlines and abstracts led to a inclusion of 8 manuscripts. Out of these 8 papers, 2 articles were excluded according to the inclusion and exclusion criteria. Finally, after full text analysis 6 manuscripts remained to be reviewed (Fig. 1).

Regarding the total of articles included (n=6), 3 were case series and 3 were clinical trials ( Table 1). The number of patients included in the selected publications was at least 5 among all articles, resulting in a mean of 17.7 patients (range: 5-51). Maxillary and mandible were treated in almost all of the studies, with the exception of Jeong *et al.* (19) who regenerated only upper jaws after sinus augmentation surgery. A total of 182 implants were placed. Regarding the type of surgery, most of the studies evaluated the effectiveness of autogenous tooth bone graft material in guided bone regeneration with the exception of Jeong *et al.* (19) who regenerated after sinus lift surgery, and Lee *et al.* (20) who recruited patients who had vertical or horizontal ridge augmentation. Concerning the grafting material used, autogenous tooth bone in a particulate form was the selected material in most of studies, eventually combined with other biomaterials. Deproteinized bovine bone mineral (DBBM) was used in 3 out of 6 studies, associated with AutoBT (19-23). Lee *et al.* (20) associated AutoBT with xenobone, allobone and synthetic bone with major defects. Analyzing the healing time span, the overall mean healing time was 4.4 months.

**Table 1:**
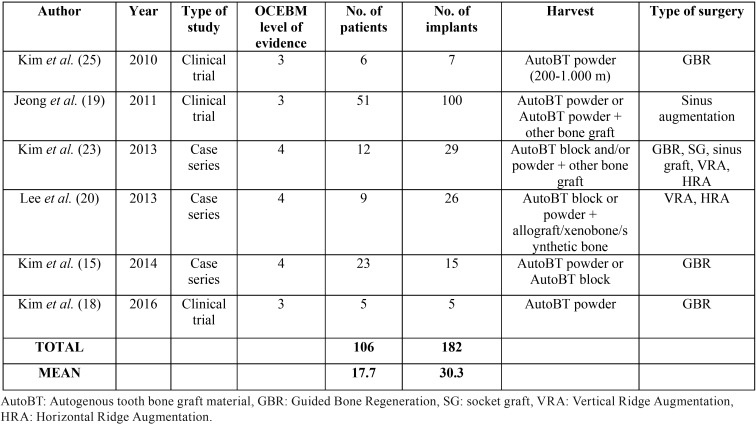
Summary of the 6 articles reviewed.

a) Evaluation of implant stability

Good stability known as the absence of clinical mobility, has long been considered as an essential factor for implant success. Some techniques have been described for implant stability measurement. In most of the studies (13,19-20) the evaluation of implant stability was evaluated using the Ostell mentor device (Integration Diagnostics AB, Sävedlen, Sweden) which uses resonance frequency analysis to measure implant mobility and stiffness, yielding the results as implant stability quotients (ISQs), which range between 1 (lowest stability) and 100 (highest) (24).

The mean primary stability of the placed implants was 67.3 implant stability quotient (ISQ), whereas the mean secondary stability was 75.5 ISQ.

b) Evaluation of complications

Clinical complications related to autogenous tooth bone regeneration were reported in  Table 2. The dehiscence of the wound was the most frequent complication. The mean wound dehiscence rate was 29.1%. In all cases, patients were all successfully treated with conservative care. Hematoma and infection developed in 2 and 5 cases, respectively.

**Table 2:**
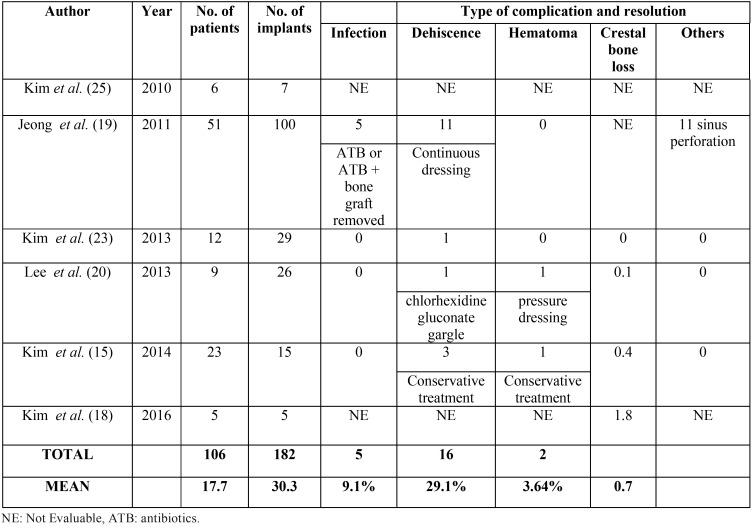
Postsurgical complications.

Three out of six studies reported crestal bone loss. The average of crestal bone loss was 0.7mm. Lee *et al.* (20) reported an average marginal bone loss after one-year loading of 0.12±0.19mm. Kim *et al.* (15) observed almost no crestal bone loss during the average 31-month follow-up period, except in 2 implants of 1 case which had crestal bone loss of 3.6 and 2.5mm. Authors attributed wound dehiscence as the cause of the bone loss. The main crestal bone loss average was reported by Kim *et al.* (18) with 1.8mm of bone loss.

c) Evaluation of implant survival and failure rates

Implant survival and failure rate were evaluated from 6 months after the prosthesis was placed. None of the reviewed studies have adopted a consistent guideline in reporting implants related data. Therefore, the assessment of implant survival rate final was limited ( Table 3). The mean abutment connection time span was 4.4 months and the follow-up average was 28.1 months. Of the 182 analyzed implants, the survival rate was 97.7% and the failure rate was 2.3% failures.

**Table 3:**
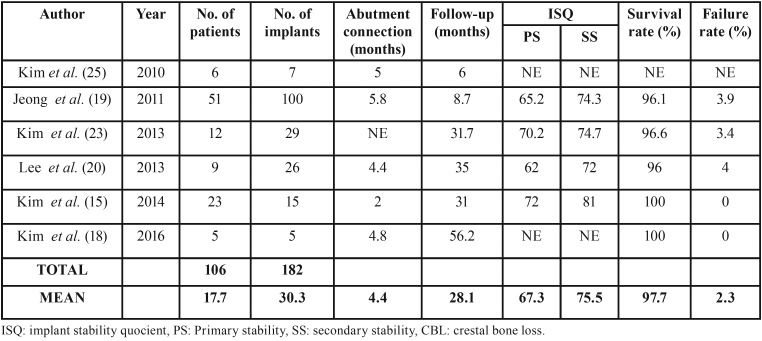
Evaluation of implant stability, crestal bone loss and implant survival and failure rates.

d) Histological and histomorphometric analysis 

All the articles reported histological and histomorphometric analysis, except Lee *et al.* (20). Kim *et al.* (25) reported that the mineral composition and histologic healing process of autogenous tooth bone graft material makes it an excellent bone graft material. They observed that new bone formation was present in 46-87% of the area of interest, during the 3-6 month healing period. Jeong *et al.* (19) observed many osteoblasts and osteoclasts surrounding the partial AutoBT, and new bone formation by process of osteoconduction and osteinduction. In the sixth month, AutoBT decreased with bone activity showing 46% to 87%. Kim *et al.* (23) performed a histopathologic examination after 2.5 months and showed excellent bone healing due to osteoconduction, showing newly formed osteoid in the reabsorbed matrix. Finally, Kim *et al.* (18) and Kim *et al.* (15) observed well-vascularized dense fibrous tissues and graft materials were directly fused with new formed bone.

## Discussion

From the analysis of the literature, few studies concerning autogenous tooth bone graft material were published. No systematic reviews or meta-analysis were found in the literature. Thus, the purpose of the this sys¬tematic review was to assemble the data reported in literature evaluating four aspects: a) implant stability, b) post-operative complication c) evaluation of implant survival and failure rates, and d) histological and histomorphometric analysis.

The topic was focused on the use of autogenous tooth bone graft material as a bone graft for ridge augmentation in both complete and partial edentulism, without any caring about the surgical protocol, considering immediate and delayed implants.

Autogenous tooth bone graft can be used in a particulate form or as a block graft. According to the literature, some study shows no significant difference in the amount of volumetric reduction between particulate bone and block bone grafts (25,26). Regarding AutoBT blocks, Kim *et al.* (23) observed in their study successful bone grafts results both in AutoBT powder in combination with blocks and AutoBT blocks alone, concluding that autogenous tooth blocks can be used as an alternative to autogenous bone blocks. Nevertheless, they did not report crestal bone loss data at any point. Curiously, Kim *et al.* (25) used AutoBT block in one patient out of 15 and they observed a crestal bone loss rate of 1mm, much higher than the average of the sample (0.47mm). On the contrary, crestal bone loss rates reported by Lee *et al.* (20) were higher in those patients grafted with powder rather than blocks.

In some protocols, AutoBT was used in combination with other bone graft materials (19,20,23). Lee *et al.* (20) used xenobone, allobone and synthetic bone all together with AutoBT in the areas with major defects, with the purpose of decrease the resorption of the graft and increase the implant stability. This is in compliance with Kim *et al.* (23) and Jeong *et al.* (19).

From the analysis of the implant stability, the ISQ average value in implant placement was 67.3 in the first surgery whereas the ISQ in the second surgery was 75.5, which was similar to the study results of Sim *et al.* (27) and Manzano *et al.* (24). In all cases, secondary stability was higher than primary stability, in terms of ISQ. Thus, it could be confirmed that when using AutoBT the implant stability increases as time passes.

Regarding the complications related to AutoBT, valuable considerations were found. Wound dehiscence was the most common complication in this surgical procedure, although favorably secondary healing was achieved after few weeks by conservative treatments (20,23). Nevertheless, Kim *et al.* (15) reported crestal bone loss rates of 3.6 and 2.5mm in 2 implants in 1 case that developed wound dehiscence. Hence, a cause-and-effect relationship between autogenous tooth graft exposure and bone loss may be deduced. Nevertheless, in all studies implant placement was always possible. Infection was another complication, exclusively reported by Jeong *et al.* (19) in which postsurgical infection developed in 5 implants. AutoBT was removed in a patient with 2 implants that developed infection, whereas the other cases resolved with antibiotics.

When analyzing the implant survival and failure rates, including an amount of 106 patients and 182 implants, the mean survival rate was 97.7% and the failure rate of 2.3%. The high survival rate could be explained by the limited number of cases treated in each study. In a recent systematic review (28), all the reported manuscripts reported a survival rate higher than 90% (range 90–100%). Survival implant rate reported in this systematic review was in accordance with the current literature (28,29).

Autogenous tooth bone graft material has been described to be an osteoconductive material with excellent biocompatibility and show high bone formation activity. Nampo et al. (7) reported that dentin contains proteins such as osteopontin (OPN) which promotes the bone formation. On immunohistochemical staining with anti-DSP antibody, the positive reaction was localized to the dentin of the extracted tooth fragments incorporated into the new bone at 6 weeks, suggesting that dentin has a high affinity for and marked osteoconductive effect on jawbone. This is in compliance with the articles reviewed. In an animal study performed by Al-Asfour *et al.* (30) human dentin graft was compared with tibia bone graft. The authors reported that demineralized xenogenic dentin onlay grafts showed similar resorption characteristics as autogenous bone onlay grafts, being resorbed in a similar rate during 12 weeks. This is in contrast with Zitzmann *et al.* (31) who reported a remaining amount of Bio-Oss of 37% at 6 months after grafting Bio- Oss.

In the study by Kim *et al.* (25) AutoBT showed gradual resorption during the first three months. At 6 months, new bone was replaced with trabecular bone with resorption of most graft material. Osteoinduction and osteoconduction were observed, which was similar to the histologic analysis of others papers (15,19,23).

This systematic review has limits because the number of articles reviewed and average sample are small. Moreover, in the current literature there are no studies that compare the efficacy of AutoBT and other typical bone graft material. Another important point is the association of variables across the included studies, such as: different teeth, anatomy considerations, methods of assessment and different types of surgeries within the same study. It is reasonable to assume that is not possible to standardize all these variables. Long-term observation research studies with bigger samples of patients should be necessary. Nevertheless, taking in account these limitations, AutoBT is considered to be useful as a bone graft material used of ridge augmentation.

Only 6 studies were included; they had limited sample sizes and short follow-up periods, and the majority was at a high risk of bias. However, it has been shown that autogenous tooth bone graft is clinically safe, has good bone forming capacity and good results are shown about implant stability.
